# Smartphone-Based Microfluidic Colorimetric Sensor for Gaseous Formaldehyde Determination with High Sensitivity and Selectivity

**DOI:** 10.3390/s18093141

**Published:** 2018-09-18

**Authors:** Xiao-Liang Guo, Yan Chen, Hong-Lan Jiang, Xian-Bo Qiu, Du-Li Yu

**Affiliations:** 1 College of Information Science and Technology, Beijing University of Chemical Technology, Beijing 100029, China; xbqiu@mail.buct.edu.cn (X.-B.Q.); dyu@mail.buct.edu.cn (D.-L.Y.); 2 Institute of Microelectronics, Tsinghua University, Beijing 100084, China; ychen2011@tsinghua.edu.cn; 3 Department of Electrical and Computer Engineering, University of Alberta, Edmonton, AB T6G 1H9, Canada; jianghonglanhit@163.com

**Keywords:** formaldehyde sensor, microfluidic chip, smartphone

## Abstract

Formaldehyde is one of the most dangerous air pollutants, which can cause sick building syndrome. Thus, it is very crucial to precisely determine formaldehyde with a low cost and simple operation. In this paper, a smartphone-based microfluidic colorimetric sensor is devised for gaseous formaldehyde determination with high sensitivity and selectivity. Specifically, a novel microfluidic chip is proposed based on the 4-aminohydrazine-5-mercapto-1,2,4-triazole (AHMT) method to determine formaldehyde; the chip consists of two reagent reservoirs, one reaction reservoir and a mixing column. In this design to prevent the fluid from flowing out while letting the gas molecule in, a hydrophobic porous poly tetra fluoroethylene (PTFE) membrane is put on the top of the reaction reservoir. Using the microfluidic chip sensor, a smartphone-based formaldehyde determination system is developed, which makes the measuring process automated and simple. As per the experiment results, the limit-of-detection (LOD) of the system is as low as 0.01 ppm, which is much lower than the maximum exposure concentration (0.08 ppm) recommended by the World Health Organization (WHO). Moreover, the sensor is hardly affected by acetaldehyde, volatile organic compounds (VOCs) or acidic-alkaline, which shows great selectivity. Finally, the performance of the proposed sensor is verified by using it for the determination of formaldehyde in a newly decorated house.

## 1. Introduction

Formaldehyde is an industrial chemical which is widely used to manufacture building materials and numerous household products, and a highly reactive compound which can be easily released into the indoor environment [1,2]. Formaldehyde is very harmful to human health and can cause irritation to the eyes and nose, central nervous system damage, immune system disorders, blindness and respiratory disease [3]. Due to its toxicity, formaldehyde is required to be determined in newly renovated houses. The World Health Organization (WHO) has set a standard for safe exposure to formaldehyde, i.e., 0.08 ppm averaged over 30 min. Thus, a high sensitivity is required for the formaldehyde sensor. Numerous kinds of interference gases often exist in houses due to human activities. Several methods have been reported for gas-phase formaldehyde determination [3,4]. Gas chromatography [4] and capillary electrophoresis [5] have high sensitivity for the determination of formaldehyde. However, these methods require expensive and laboratory-based apparatus, thus it is impossible to use these methods for formaldehyde determination in personal houses [3]. The methods based on metal oxides [6,7,8,9,10,11,12] and piezoelectric sensors [13,14,15] have a small size and low cost. However, they are mostly interfered with by toluene, alcohol, acetone and other volatile organic compounds (VOCs), which indicates poor selectivity. Therefore, high selectivity for the formaldehyde sensor is also very important. Enzyme-based biosensors [16,17] have been studied because of their high selectivity by using the formaldehyde dehydrogenase.

Spectrophotometric methods have also been intensively investigated for formaldehyde determination using various sensing reagents such as chromotropic acid [18,19], pararosaniline [20], 3-methyl-2-benzothiazolone hydrazine (MBTH) [21], 4-amino-3-penten-2-one (fluoral-p) [22,23], 2,4-dinitrophenylhydrazine (DNPH) [24], β-diketone [25,26,27] and 4-aminohydrazine-5-mercapto-1,2,4-triazole (AHMT) [28]. Chromotropic acid, pararosaniline, MBTH and fluoral-p are, however, prone to be interfered with by acetalydehyde [29] or provide poor limits of detection [22,30], while β-diketone [25] and AHMT could have high selectivity and sensitivity. Formaldehyde-sensing elements by incorporating β-diketone in porous glasses [25,31], sol-gel strips [3] and paper strips [27] have been developed with high sensitivity and selectivity. However, some of these methods have a long sampling time from more than one hour to 24 h.

Due to its high selectivity and sensitivity as well as a relatively short sampling time [28], AHMT is used in this work for formaldehyde determination in a personal environment. Its low cost, small size and simple operation are also very important. Thus, a novel microfluidic chip is proposed. The chip consists of two reagent reservoirs, one reaction reservoir and a mixing column. In order to prevent the fluid from flowing out but let the gas molecule in, a hydrophobic porous poly tetra fluoroethylene (PTFE) membrane is used on the top of the reaction reservoir. Based on the microfluidic chip, a measuring sensing system is developed for automatically detecting the formaldehyde. With their increasing development, smart mobile devices are usually equipped with excellent digital color cameras which are available for evaluating the color of an object. Therefore, in this paper, a smartphone is used as the color sensor and the controller of the sensing system, which contributes to reducing the cost and simplifying the operation greatly. A simple smartphone application is developed for the automatic determination of the formaldehyde. The limit-of-detection (LOD) of the sensing system is as low as 0.01 ppm. Moreover, the experiment results show that the sensor is hardly affected by acetaldehyde, VOCs or acidic-alkaline, which shows great selectivity. The effects of temperature and humidity are also studied. Considering the above advantages such as high sensitivity, high selectivity, low cost, small size (5 cm × 5 cm × 10 cm), simple operation and a relatively short sampling time, this smartphone-based microfluidic formaldehyde sensor is very suitable for personal prevention and the control of sick building syndrome. Finally, the concentrations of formaldehyde content in a newly decorated house are determined using the proposed sensor.

## 2. Experimental Section

### 2.1. AHMT Method

In this design, 0.1 g AHMT was dissolved in 10 mL of 9% hydrogen chloride (HCl) solution, and 2.3 g potassium hydroxide (KOH) was dissolved in 10 mL of water; they are referred to as reagent solutions 1 and 2, respectively. The mixture of reagent solutions 1 and 2 (1:1) could react with formaldehyde, and the color of the solution would change to purple [32]. The shade of the color relates to the amount of absorbed formaldehyde. The AHMT, HCl and KOH used in this experiment are from Sinopharm Chemical Reagent Co., Ltd. (Beijing, China). The reagents were of analytical reagent grade and were used as received. The measurements were carried out at 20 ± 1 °C, unless stated otherwise.

### 2.2. Standard Gaseous Formaldehyde

Aqueous formaldehyde could be used to generate equilibrium concentrations of gaseous formaldehyde according to the equation below [33]:

(1) $$\left[\mathrm{HCHO}\left(\mathrm{aq}\right)\right]=16650{\left[\mathrm{HCHO}\left(g\right)\right]}^{1.0789}\;$$

A range of diluted formaldehyde solutions were placed in 50 mL polypropylene exposure tubes to give the specified equilibrium concentrations of gaseous formaldehyde. Thus, standard gaseous formaldehyde was obtained. The relationship between the concentration of diluted formaldehyde aqueous solution and gas-phase formaldehyde concentration is shown in Table S1.

### 2.3. Microfluidic Chip Configuration

**Figure 1:**
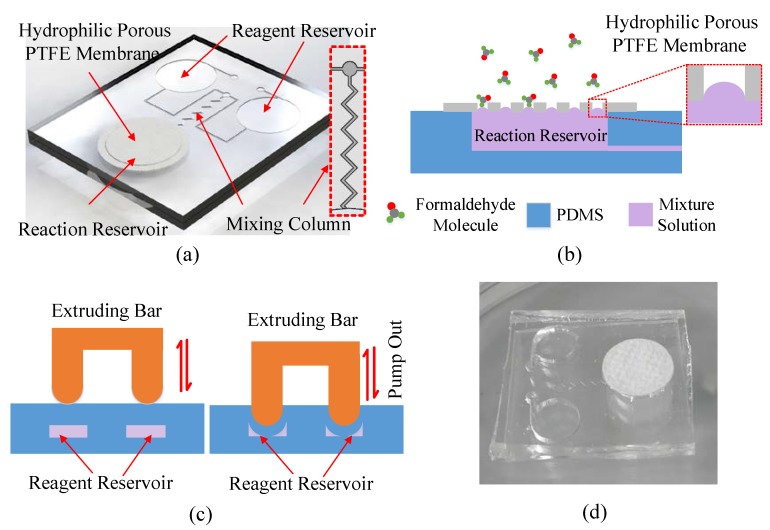
(**a**) Three-dimensional diagram of a microfluidic chip, (**b**) the working principle of the reaction reservoir, (**c**) the working principles of the reagent reservoirs and mixing, (**d**) the image of the microfluidic chip.

Based on the AHMT method, a novel microfluidic chip was developed. Figure 1a illustrates the major components of the proposed microfluidic chip, the mixing column, the reagent reservoirs for containing solution A and solution B, the reaction reservoir for the mixture reacting with formaldehyde and the hydrophobic porous PTFE membrane. The microfluidic microchip was made by bonding two 10 mm × 20 mm molded polydimethylsiloxane (PDMS) substrates together with the oxygen plasma using plasma surface treatment equipment (Europlasma CD400, Belgium; Power: 230 W; Vacuum: 13 Pa with a flow of 20 mL/min). The bottom PDMS substrate with a thickness of 2 mm consisted of the microfluidic channels with a height of 50 μm and a width of 500 μm, while the top PDMS substrate with a thickness of 2 mm consisted of three cylindrical reservoirs with radiuses of 8 mm. The heights of the two reagent reservoirs were 1 mm, and the height of the reaction reservoir was 2 mm. That is to say, the reaction reservoir was a through-hole on the top substrate. Following the bonding step, a hydrophobic porous PTFE membrane was put on the top of the reaction reservoir. The mixing column featured a zigzag channel with a 90° angle and a period step of 2 mm. The effective length of the mixing channel is 16 mm.

The microfluidic chip fabrication and assembly are detailed in Figure S1. The two reagent reservoirs were filled with solution A and solution B, respectively (the filling step is shown in Figure S2). The reaction reservoir was empty until solution A and solution B were driven into the reaction reservoir by extruding the reagent reservoirs, which is called the pumping out action (right part Figure 1c). When the extruding actuators were up, the mixture solution went back to the reagent reservoirs, which is called the pumping in action. Due to the fact that the microfluidic channel was at the bottom of the reservoirs, bubbles were hardly generated by the pumping action. Solution A and solution B were thoroughly mixed by pumping in and out several times (as shown in Figure 1c). The mixture was finally driven into the reaction reservoir to react with the formaldehyde. As the porous membrane is hydrophobic, the solution could hardly flow out (Figure 1b), which is very important for personal use. Even when 20 kPa pressure was further applied to the solution, the solution could still not flow out. (The testing method is shown in Figure S3). However, the formaldehyde molecules can get in freely through the micropores on the membrane; the working principle is shown in Figure 1b. If the concentration of formaldehyde is high, many molecules will enter the reaction reservoir and react with the reagent. Therefore, the color change will be more obvious than that under low concentration of formaldehyde within the same length of time. Thus, a disposable gaseous formaldehyde sensor based on the microfluidic chip was finally developed. Figure 1d shows a photograph of the disposable gaseous formaldehyde sensor.

### 2.4. Smartphone-Based Formaldehyde Determination System

**Figure 2:**
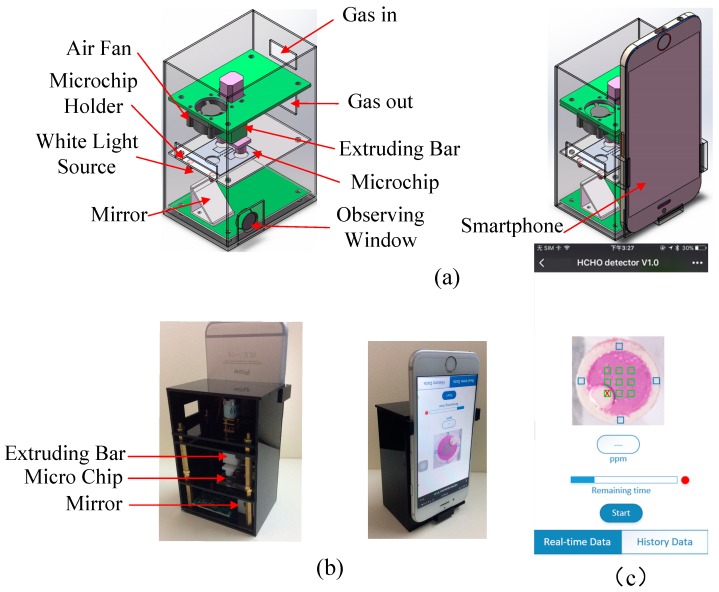
(**a**) The diagram of the smartphone-based formaldehyde determination system. (**b**) The photographs of the smartphone-based formaldehyde determination system. (**c**) The user interface of the app.

As shown in Figure 2a, based on the microfluidic chip, a smartphone-based formaldehyde determination system was developed for personal house formaldehyde determination. It comprised a microfluidic chip, a smart phone for capturing the images, an extruding bar controlled by a linear microactuator for extruding the reagent reservoirs, a fan for air ventilation (its flow rate is 28.3 L/min), a flat mirror and a white light source. The microfluidic chip can be easily placed into the microchip holder. The microchip holder confines the sensor to a relatively fixed position, which is very important for the following image processing. The flat reflective surface of the mirror orientated at 45° folds the optical path to make the system more compact. The smartphone was orientated on the observing window such that the bottom of the reaction reservoir was within the field of view of its digital camera. Figure 2b shows the photographs of the smartphone-based formaldehyde determination system. Its housing is made of black polymethyl methacrylate (PMMA), which can shade the interference light outside.

**Figure 3:**
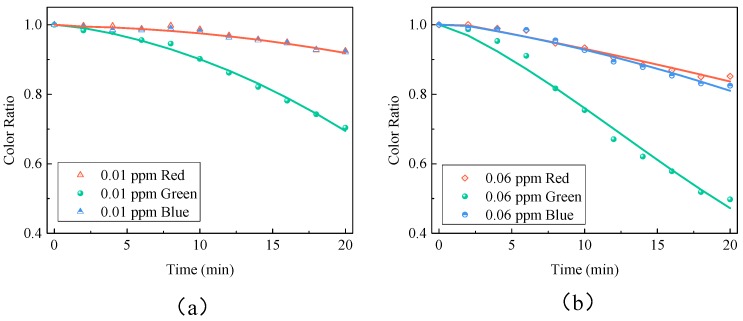
Changes in color ratios of red, green and blue over time in the presence of (**a**) 0.01 ppm and (**b**) 0.06 ppm formaldehyde, respectively.

Moreover, a smartphone application on IOS was developed using Xcode and the user interface of the application is shown in Figure 2c. In the center of the interface of the application, there is an image window (2 cm × 2 cm) which shows the reaction reservoir. Under the image window, a text box is used to display the final concentration when one measurement was finished. A time bar was also designed for indicating the remaining time of the measurement. On the right of the time bar, there is an indicator showing whether the application is busy or not. The start button at the bottom of the interface is used to start a measurement. Before use, the smartphone should be connected with the system via Bluetooth. When placing the microfluidic chip on the holder, we needed to open the application, press the start button and then place the smart phone on the phone holder. The app would start the extruding bar motor to make the bar go up and down three times to mix solutions A and B. Meanwhile, the fan was started to sample the fresh air. The time was precisely controlled by the application, which could increase the repeatability of the determination results. During the following 20 min, the images of the color changing could be obtained. To get a precise result, two kinds of regions of interest on the image were assigned on the screen of the smart phone (Figure 2c), which were referred to as the reference region and the reaction region. As shown in Figure 2c, the reference regions are marked by blue boxes. The four reference regions were located outside the circle of the reaction reservoir but inside the circle of the white hydrophobic porous PTFE membrane. The nine reaction regions (marked by green boxes in Figure 2c) were located inside the circle of the reaction reservoir and organized into a 3 × 3 array. The color ratios of the nine reaction regions are calculated according to the formula below:

(2) $$Cr_{i}=\frac{Crea_{i}}{Cref_{i}}\;\left(i=\mathrm{Red},\mathrm{Green},\mathrm{Blue}\right)\;$$

where *Cr_i_* refers to the color ratio of red, green or blue, while *Crea_i_* and *Cref_i_* are the average color values of each reaction and reference region, respectively, for minimizing the effect of the tiny bubbles. Nine sets of data were then obtained for nine reaction regions. However, some of them might be invalid because of the air bubbles, as shown in Figure 2c. The invalid data could be identified by sorting the data from the largest to the smallest and calculating the differences between adjacent data. Then, the invalid regions were marked with a red cross. The average color ratio of the valid data was calculated as the final color ratio. Figure 3 shows the color ratios of red, green and blue. It shows that the color ratio of green changes the most with time. Thus, the color ratio of green can be used as the most valid indicator of the formaldehyde concentration.

## 3. Results and Discussion

### 3.1. Relationship between Sampling Time and Color Ratios

**Figure 4:**
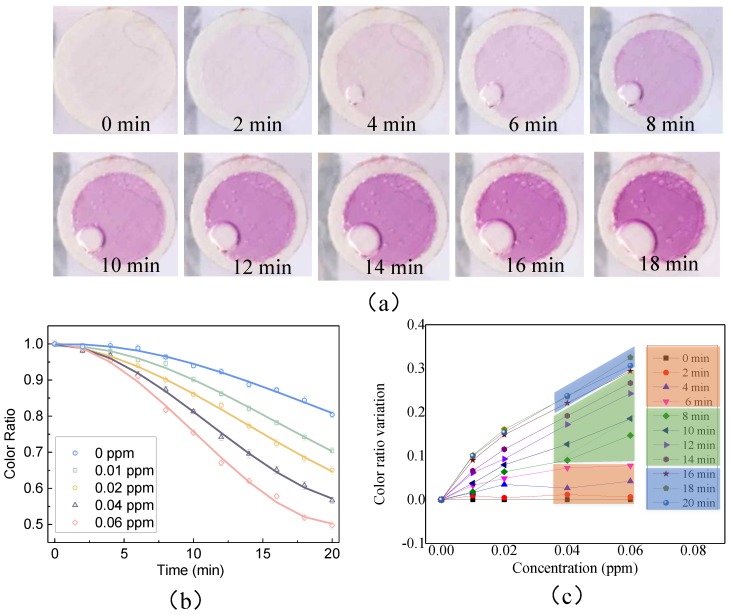
(**a**) Photographs of the reaction reservoir at different times. The concentration of the formaldehyde is 0.06 ppm. (**b**) Relationship between sampling time and color ratios at various concentrations of formaldehyde. (**c**) The color ratio variation relating to the color ratio at 0 ppm.

Figure 4a shows the resultant color changes with time when using the proposed sensor to determine formaldehyde with a concentration of 0.06 ppm. Over time, the color becomes darker. Figure 4b shows the relationship between the sampling time and the color ratio at various concentrations of formaldehyde. The color intensity increases with the increase of the sampling time. The color ratio variations from the color ratio at 0 ppm were calculated. The relationship between the color ratio variation and the concentration at different times was plotted (as shown in Figure 4c). We can see that the concentrations could hardly be distinguished (marked by orange) within the first 6 min. Between 8 and 14 min, the resolution of the concentrations starts to increase sharply with the sampling time. After 16 min, the resolution of the concentrations becomes almost stable. Thus, the 18 min was chosen as the optimal sampling time and about 509.4 L of air flew over the device for each test.

### 3.2. Calibration of Smartphone-Based Microfluidic Colorimetric Sensor for Gaseous Formaldehyde Determination

**Figure 5:**
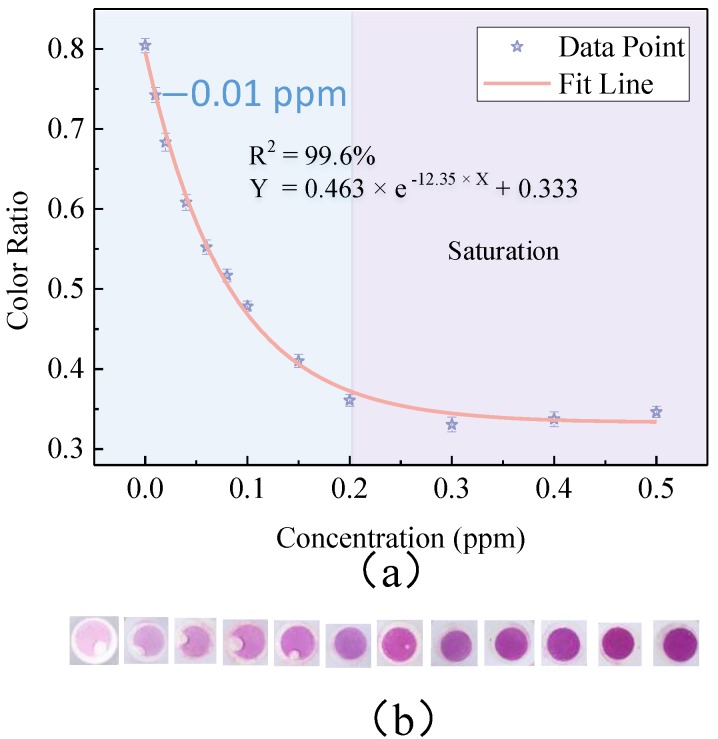
(**a**) Calibration curve of smartphone-based microfluidic colorimetric sensor for gaseous formaldehyde determination. (**b**) The corresponding reaction reservoir images of different concentrations.

A calibration curve was obtained over a concentration range of 0–0.5 ppm, as shown in Figure 5. Figure 5b shows the corresponding reaction reservoir images of the measured concentrations of formaldehyde. The exponential function was used to fit the data, and the fitting equation is given by

(3) $$\mathrm{Color}\;\mathrm{Ratio}=0.463\times e^{\left(-12.35\times \mathrm{Con}\right)}+0.333\;$$

where Con refers to the concentration of formaldehyde. The adjusted R^2^ is 0.996, which indicates an excellent fitting result. Three measurements were conducted for each concentration of formaldehyde and the standard deviation of the three measurements for each concentration was calculated, which show the repeatability of the analyzer. Figure 5 shows that the color ratio decreases with the increase of the concentration when the concentration is below 0.2 ppm. However, the color ratio is hardly changed when the concentration is higher than 0.2 ppm. Therefore, the detection range of the sensor can be concluded as 0–0.2 ppm, which covers the WHO standards (0.08 ppm). The limit-of-detection (LOD) of our sensor was 0.01 ppm. Thus, this method can be used to detect formaldehyde at WHO standards in air within 20 min. A table for comparison with other methods based on the AHMT method is shown in Table 1. We can see that the microfluidics-based method had a lower LOD than others and the sampling time was moderate, but still very short.

### 3.3. Selectivity of the Smartphone-Based Microfluidic Colorimetric Sensor

**Figure 6:**
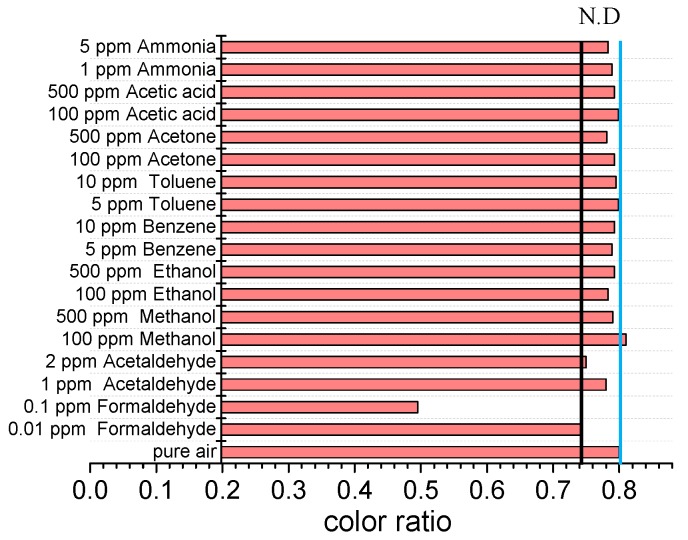
The color ratio of different kinds of gases.

The selectivity of the sensor was verified by using acetaldehyde, VOCs and acidic-alkaline with different concentrations. The final color ratios were recorded and shown in Figure 6. The black line refers to the color ratio of 0.01 ppm formaldehyde while the blue line refers to the color ratio of the pure air. A gas could be considered as undetectable if its color ratio is between the two lines. As per Figure 6, only formaldehyde is detected among the considered gases. Therefore, the sensor is hardly affected by the presence of the other gases, which proves the selectivity of the sensor for formaldehyde. 

### 3.4. Effect of Temperature and Humidity

**Figure 7:**
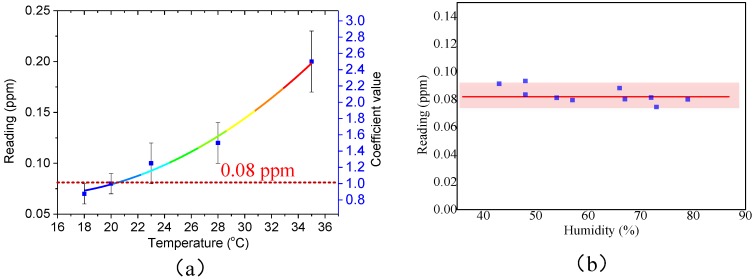
(**a**) The relationship between the reading of the calibrated system and the temperature in the presence of 0.08 ppm formaldehyde. The red dot line refers to the 0.08 ppm under 20 °C. The blue y-axes on the right show the corresponding coefficient values. (**b**) The blue dots show the reading at a different relative humidity from 40% to 80%. The light red box shows the range of the reading.

Figure 7a shows the effect of the temperature variation for the proposed sensor in the range of 18–35 °C. We can see that the coefficient value (the ratio of the reading under a certain temperature to that under 20 °C) increases with the increase of the temperature. Therefore, the final result must be corrected when the temperature is not 20 °C. The humidity in the gas was controlled in the range of 40–80% RH at 20 °C, and the final result is shown in Figure 7b. We can see that the response to formaldehyde is hardly affected by humidity in the examined range. 

### 3.5. Determination of Formaldehyde in a Newly Decorated House

**Figure 8:**
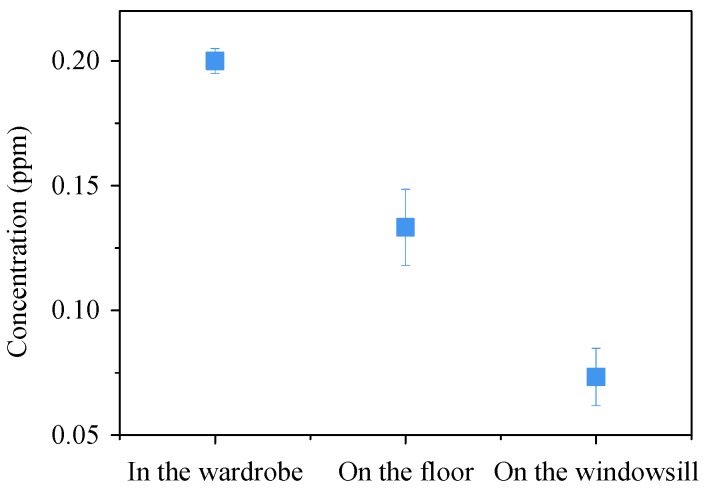
The formaldehyde determination results in a newly decorated house.

The smartphone-based microfluidic colorimetric sensor was evaluated for the determination of formaldehyde in a newly decorated house. Three places—inside the wardrobe, on the floor and on the windowsill—were selected for the determination. For each place, three smartphone-based microfluidic colorimetric sensors were employed simultaneously. The house and wardrobe were closed for 24 h before conducting the measurements. During the measurement, the temperature was controlled to be around 20 °C by using an air conditioner. The results reported in Figure 8 demonstrate that the concentrations of formaldehyde on the windowsill and on the floor are 0.07 ppm and 0.13 ppm, respectively, while the concentration in the wardrobe is above 0.2 ppm saturated. It also shows that the sensors have a good consistency between individual samples of the same batch (relative standard deviations were below 16%).

## 4. Conclusions

In this paper, a formaldehyde gas sensor was proposed based on a novel microfluidic chip and a smartphone for the prevention and control of sick building syndrome. The novel microfluidic chip is designed based on the ATHM method; it consists of two reagent reservoirs and one reaction reservoir. In order to prevent the fluid from flowing out, a hydrophobic porous PTFE membrane was used on the top of the reaction reservoir. Meanwhile, the gas molecule can get in and out freely. The performance of the sensor has been evaluated. We conclude that the smartphone-based microfluidic colorimetric sensor for gaseous formaldehyde determination has a very low LOD (0.01 ppm), which is much lower than the maximum exposure concentration recommended by the WHO (0.08 ppm). The sensor is also hardly affected by acetaldehyde, VOCs or acidic-alkaline, which shows great selectivity. Additionally, the sensor has the advantages of being low-cost, small-sized and simple to operate, which makes it very suitable for personal indoor formaldehyde determination.

## Figures and Tables

**Table 1:** A comparison of the AHMT-based methods for formaldehyde determination.

Method	LOD (ppm)	Sampling Time (min)
Petri dish-based method [34]	<0.015 ^1^	24 (h)
Paper-based method [28]	0.04	3
Microfluidics-based method (this study)	0.01	18

^1^ The data were calculated based on Figure 5 in Reference [34].

## Supplementary Materials

The following are available online at http://www.mdpi.com/1424-8220/18/9/3141/s1, Figure S1: the fabrication process. Figure S2: the process of sealing reagent in the microfluidic chip. Figure S3: testing the ability a hydrophilic porous PTFE membrane to prevent water from flowing out. Figure S4: the diagrams of the mixing step using the pumping. Table S1: The relationship between the concentrations of the aqueous formaldehyde solution and equilibrate gaseous formaldehyde at 20 °C.
