# Supplementary material for: Smartphone-Based Microfluidic Colorimetric Sensor for Gaseous Formaldehyde Determination with High Sensitivity and Selectivity
Source: Sensors (Basel). 2018 Sep 18;18(9):3141. doi: 10.3390/s18093141 (PMC6165092; doi:10.3390/s18093141)
Supplement: Supplementary file 1 [file sensors-18-03141-s001.pdf]

Supporting Information

Smartphone-Based Microfluidic Colorimetric Sensor for Gaseous Formaldehyde Determination with High Sensitivity and Selectivity

Xiao-Liang Guo [1,*,†], Yan Chen [2,†], Hong-Lan Jiang [3], Xian-Bo Qiu [1] and Du-Li Yu [1]

[1] College of Information Science and Technology, Beijing University of Chemical Technology, Beijing 100029, China; xbqiu@mail.buct.edu.cn (X.-B.Q.); dyu@mail.buct.edu.cn (D.-L.Y.)
[2] Institute of Microelectronics, Tsinghua University, Beijing 100084, China; ychen2011@tsinghua.edu.cn
[3] Department of Electrical and Computer Engineering, University of Alberta, Edmonton, AB T6G 1H9, Canada; jianghonglanhit@163.com
* Correspondence: gxl@mail.buct.edu.cn
† These authors contributed equally to this work.

Part 1

Table S1. The relationship between the concentrations of the aqueous formaldehyde solution. and equilibrate gaseous formaldehyde at 20 °C.

Concentration of aqueous HCHO (mol L^{-1})	Concentration of gaseous HCHO (ppm)
3.89×10^{-5}	0.01
8.22×10^{-5}	0.02
1.74×10^{-4}	0.04
2.69×10^{-4}	0.06
3.67×10^{-4}	0.08
4.67×10^{-4}	0.10
7.23×10^{-4}	0.15
9.86×10^{-4}	0.20
1.50×10^{-3}	0.30
2.10×10^{-3}	0.40
2.60×10^{-3}	0.50

Part 2. Fabrication

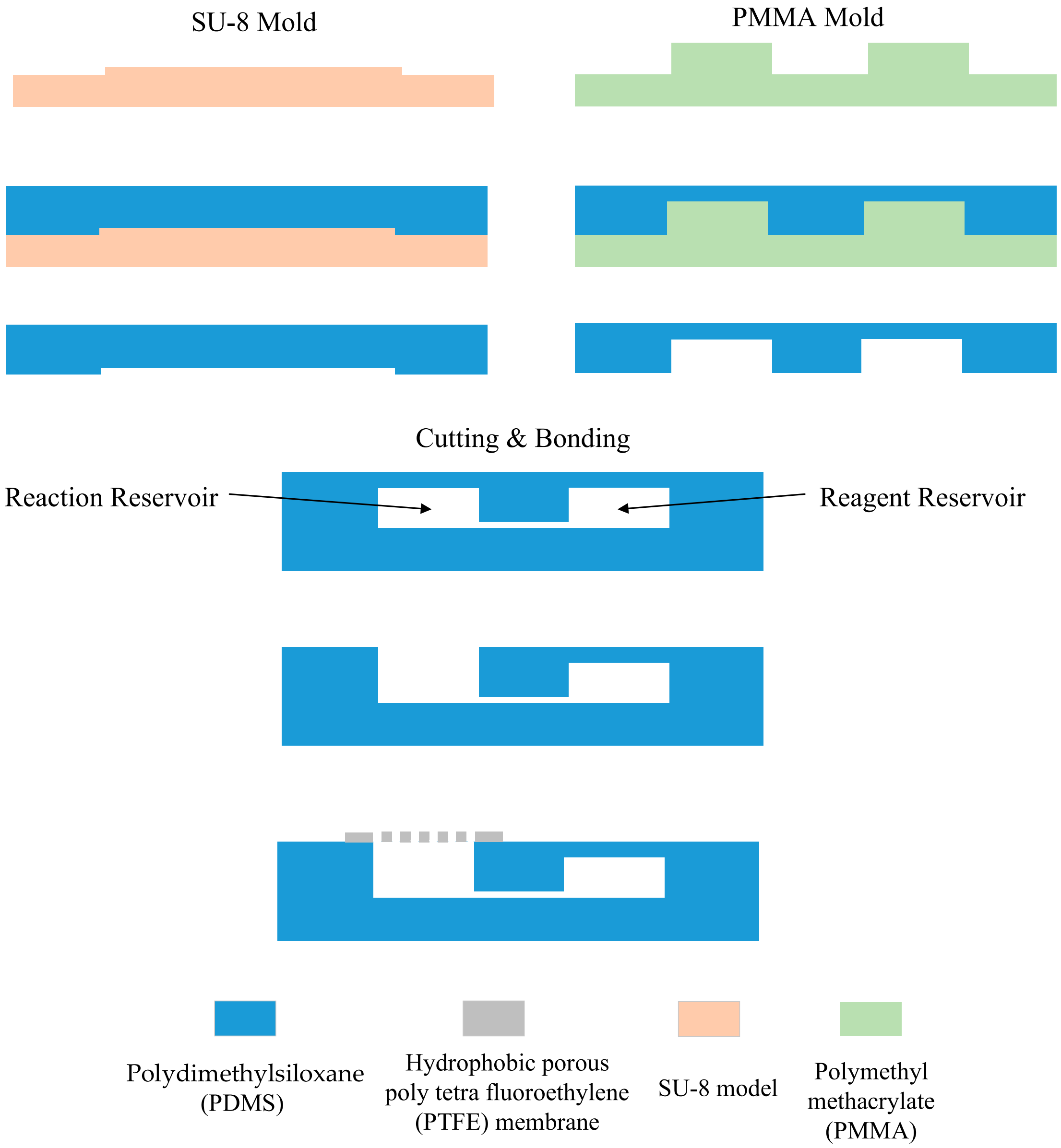

Figure S1. The fabrication process.

First, the two parts of polydimethylsiloxane (PDMS), cross-linker/curing agent A and siloxane B, were mixed in a 1:10 ratio. Upon mixing, the PDMS was degassed in a vacuum chamber to remove any bubbles. Two kinds of PDMS casting molds were fabricated. One was made by machining a polymethyl methacrylate (PMMA) board; the other one was made by means of lithography using SU-8 resist. The casting process was as simple as placing the mold in a heat-tolerant plastic tray and pouring PDMS onto the molds. The thickness of the PDMS was controlled to be 2 mm. Placing the resulting PDMS in an oven at 95 °C for 1 h cured the PDMS, which was then peeled away from the mold and cut into a rectangle of 10 mm × 2 mm. In order to bond the two parts together to form the final microfluidic chip, they were treated with oxygen plasma for 2 min using plasma surface treatment equipment (Europlasma CD400, Belgium; Power: 230 W; Vacuum: 13 Pa with a flow of 20 ml/min). To aid with alignment, a

small amount of methanol was used to keep the surfaces separate for a short time. After bonding, a hole was drilled on the reaction reservoir. Finally, a hydrophilic porous poly tetra fluoroethylene (PTFE) was pasted on the hole.

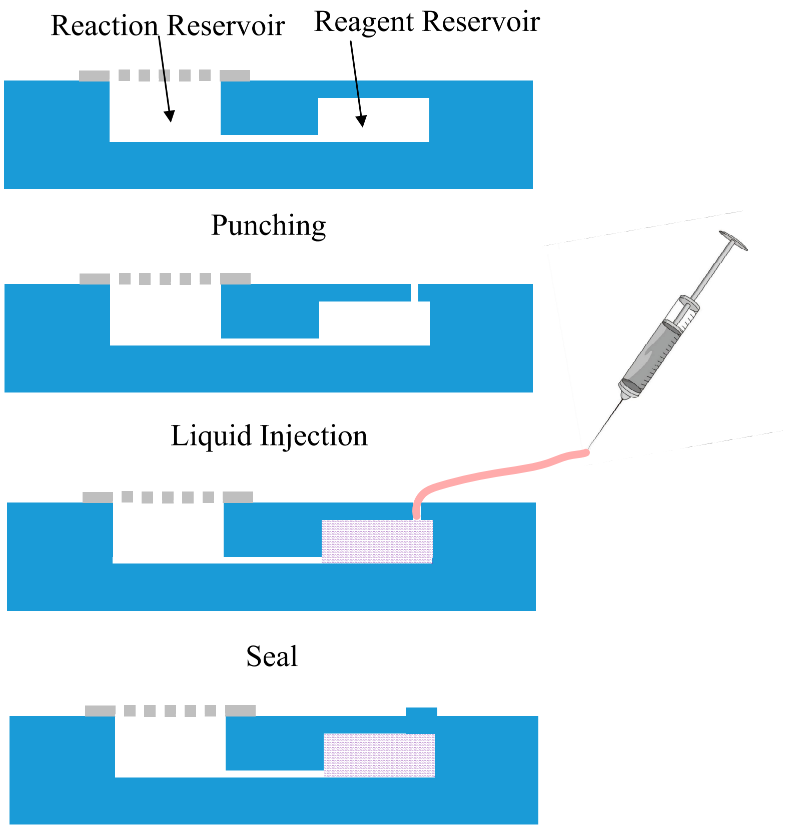

Figure S2. The process of sealing reagent in the microfluidic chip.

Firstly, a tiny hole is made in the reagent reservoir. Secondly, the reagent is injected into the reservoir through the hole using an injection syringe. Because of the hydrophobic property of the micro-channel, the reagent can hardly go into the reaction reservoir if the injecting speed is low. Thirdly, a tiny drop of PDMS is dropped onto the hole. The chip is then put in a plastic petri dish and the dish is sealed using parafilm. After 24 h at room temperature, the PDMS on the hole can be solidified, and the chip is finally obtained.

Part 3. Hydrophilic Porous PTFE Membrane Testing

A hydrophilic porous PTFE membrane was firstly put on the bottom of a plastic pipe. Then, the water was injected into the pipe. The initial height of the water was 10 cm. The water leakage was tested after 30 min. The height of the water was then increased with a step of 10 cm until the water leaked. The pressure applied to the membrane (P) was calculated using the following equation:

$$P = \rho g h$$

where h is the height of the water, ρ is the density of the water and g is the acceleration of gravity.

The final height of the water obtained was 200 cm, which means that even when 20 kPa

pressure was further applied to the solution, the solution could still not flow out.

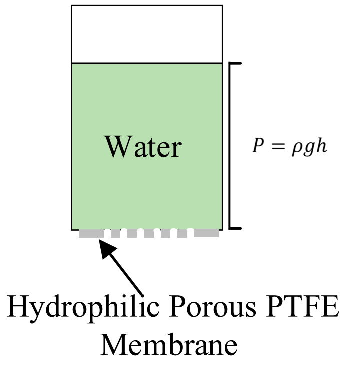

Figure S3. Testing the ability of a hydrophilic porous PTFE membrane to prevent water from flowing out.

Part 4. The Mixing Process

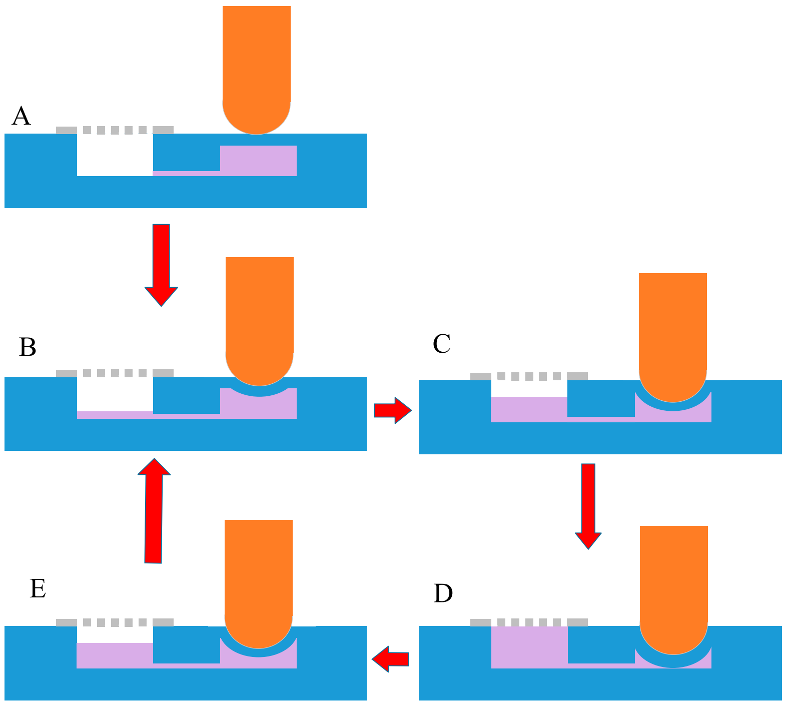

Figure S4. Diagrams of the mixing step using pumping. At the beginning, the extruding bar is moving slowly down (**A**, **B**, **C**). Then the extruding bar is moving up (**D**, **E**, **B**). The mixing is realized by repeating the steps of **B-C-D-E-B**.

A, B and C show the extruding bar moving slowly down from the initial situation and no bubbles being generated due to the fact that the reagent reservoirs are filled with solution without air. D, E and B show that the extruding bar is moving up and no bubbles are being generated due to the fact that the microfluidic channel was at the bottom of the reservoirs and immerged in the solution.
